# *Fagonia indica* Repairs Hepatic Damage through Expression Regulation of Toll-Like Receptors in a Liver Injury Model

**DOI:** 10.1155/2018/7967135

**Published:** 2018-07-02

**Authors:** Fareeha Azam, Nadeem Sheikh, Gibran Ali, Asima Tayyeb

**Affiliations:** ^1^Department of Zoology, University of the Punjab, Lahore, Pakistan; ^2^School of Biological Sciences, University of the Punjab, Lahore, Pakistan; ^3^Centre of Excellence in Molecular Biology, Lahore, Pakistan

## Abstract

*Fagonia indica* is a traditionally used phytomedicine to cure hepatic ailments. However, efficient validation of its hepatoprotective effect and molecular mechanisms involved are not yet well established. Therefore, the present study was designed to evaluate the hepatoprotective activity of *Fagonia indica* and to understand the molecular mechanisms involved in the reversal of hepatic injury. The liver injury mouse model was established by thioacetamide followed by oral administration of plant extract. Serum biochemical and histological analyses were performed to assess the level of hepatic injury. Expression analysis of proinflammatory, hepatic, and immune regulatory genes was performed with RT-PCR. Results of serological and histological analyses described the restoration of normal liver function and architecture in mice treated with plant extract. In addition, altered expression of proinflammatory (IL-1*β*, IL-6, TNF-*α*, and TGF-*β*) and hepatic (krt-18 and albumin) markers further strengthens the liver injury reversal effects of *Fagonia indica*. Furthermore, a significant expression regulation of innate immunity components such as toll-like receptors 4 and 9 and MyD-88 was observed suggesting an immune regulatory role of the plant in curing liver injury. In conclusion, the current study not only proposes *Fagonia indica*, a strong hepatoprotective candidate, but also recommends an immune regulatory toll-like receptor pathway as an important therapeutic target in liver diseases.

## 1. Introduction

The liver, being the first filter organ for toxic chemicals and imprudent metabolites, plays a vital role in the acquisition of normal homeostasis. Detoxification of toxins, therefore, renders it highly susceptible to tissue injuries and cellular death [1]. The liver with its remarkable immune-tolerance mechanism [2] and regenerative capacity can efficiently deal with minor hepatic insults [3, 4]. Nonetheless, improper detoxification of chemical hepatotoxicants is a serious issue. It accounts for about 50% of all acute liver failure (ALF) cases [5]. An acute or chronic hepatic injury involves recognition of pathogen-associated molecular patterns (PAMPs) and damage-associated molecular patterns (DAMPs) by toll-like receptors (TLRs) [6]. TLRs are an important class of pattern recognition receptors and a component of the innate immune system [7]. Prolonged or repeated hepatic injury results in a complex interplay of hepatocytes, Kupffer cells, natural killer cells, hepatic stellate cells (HSCs) [8, 9], dendritic cells (DCs), and liver sinusoidal endothelial cells (LSECs). These cells together play an overall immune-suppressive role in the liver [10]. Kupffer cells and HSCs are the main hepatic cell types that respond to PAMPs and DAMPs by TLR expression [6]. TLR stimulation results in the activation of proinflammatory pathways involving chemokines, cytokines, complement proteins, acute phase proteins, and death ligands [11, 12]. Proinflammatory cytokine transcription starts within just a few minutes after a PAMP/DAMP exposure [13]. The significance of TLR signaling pathway activation in various hepatic diseases such as inflammation, fibrosis, immunity, and tumorigenesis has rendered it an important therapeutic target [14].

Inflammation and wound healing are interconnected processes where inflammatory signals compel immune cells towards the site of injury [15]. Consequently, repair and regeneration of injured tissue occur via apoptotic and regenerative mechanisms [16]. Tissue scarring and accumulation of extracellular matrix are hallmarks of inflammation. However, in chronic injury, the wound healing process becomes maladaptive leading to the loss of functional hepatic parenchyma. This condition ends up with liver fibrosis, which might lead towards hepatic cirrhosis and carcinoma [7, 17–22]. So far, drugs used for hepatic injury treatment are incapable of complete reversal of cirrhosis though results of certain clinical trials report their antifibrotic therapeutic potential [23].

Medicinal plants are gaining popularity due to their versatility, safety, and cost-effectiveness. In view of drug-induced hepatotoxicities, use of phytomedicine as antifibrotic agent is on the rise nowadays. Many medicinal plants have shown antifibrotic activity by targeting immunity and inflammation [24]. Genus *Fagonia* has been studied for its medicinal significance against a broad range of diseases. Locally, it is called by the name “Dhamasa” in the Indian subcontinent [25]. Several members of the genus have been reported for hepatoprotective activity along with many other important medicinal activities. *Fagonia cretica* has been described for hepatoprotective [26], antipyretic [27], antidiabetic [28, 29], and hematological [30] properties. *Fagonia arabica* has been reported for its thrombolytic [31] and antioxidant activities [32]. Few other members such as *Fagonia schweinfurthii* and *Fagonia bruguieri* have revealed anti-inflammatory and antioxidant activities against hepatic injury [28, 33]. Similarly, the protective activity of *Fagonia olivieri* has been claimed against hepatic and hepatorenal injury in rat models [34, 35].

*Fagonia indica* is one of the important members of genus *Fagonia*. This thorny herb is known locally as “Dhaman” and “Sacchi Booti” with an approximately 60 cm height and 100 cm width [36] growing widely in Asian and African deserts [37]. It is a small, green undershrub distributed largely in Afghanistan, Egypt, and calcareous rocks of Western India and Pakistan [38]. Traditionally, it has been used for antipyretic and anti-inflammatory effects [39]. An aqueous decoction of aerial parts of this plant is used to induce abortion [40] and as a remedy to cure cancer at early stages [41, 42]. Previously, it has been described for its analgesic [43] and anticancer activities [44]. A recent study has described the protective activity of *Fagonia indica* against gastric ulcer [45]. Preliminary studies have also reported the hepatoprotective effect of *Fagonia indica* [46]. However, mechanisms of its action and the molecular pathways involved are still not explored. Therefore, the aim of the present study was to investigate molecular mechanisms involved in the hepatoprotective activity of *Fagonia indica*. A thioacetamide- (TAA-) induced hepatic injury mouse model was used. Our findings highlighted the hepatoprotective potential of *Fagonia indica* through regulation of inflammatory and innate immunity-related TLR pathways.

## 2. Methodology

### 2.1. Plant Collection and Preparation of Plant Extract

*Fagonia indica* was collected fresh from Pind Dadan Khan Tehsil, a subdivision of District Jhelum, Punjab, Pakistan. The plant was identified by Department of Botany, University of the Punjab, Lahore, Pakistan. The collected *Fagonia indica* was dried under shade, in a relatively dark area. The dried whole plant material was powdered using a dry grinder considering smaller particle size better for efficient solvent extraction. Ethanolic (in 70% ethanol) extract was prepared from the plant using a standard plant extract preparation (maceration) protocol [47]. Briefly, 20 g plant material was suspended in 200 mL solvent (in 1 : 10 *w*/*v* ratio) for three days with constant agitation. After three days, the solution was filtered and solvents were evaporated at room temperature. The dried extract was stored at −20°C until use.

### 2.2. Animals

Swiss albino male mice reared in an animal house of the School of Biological Sciences were used for this study. During the study, animals were given free access to water and food pellets while the room temperature was maintained between 23 and 26°C. All animals received humane care. Animal handling guidelines devised by the ethical society of University of the Punjab were followed for all experiments.

### 2.3. Acute Toxicity Test for Plant Extract

Male mice weighing between 26 and 34 g were divided into five groups (*N* = 8) for determination of acute toxicity of *Fagonia indica*. LD_50_ of the plant extract was estimated using 50% death within 72 hr following oral PE administration at different doses. During this time interval, number of animal deaths was expressed in percentile. The probit test was applied to determine LD_50_ by using percent deaths per group versus doses' log [48].

### 2.4. Liver Injury Mouse Model

TAA is a hepatotoxicant widely used in acute and chronic liver injury studies [49] and to understand fibrogenic mechanisms [50, 51]. In order to develop a hepatic injury model, a 100 mg/kg body weight dose of TAA (Fluka catalogue number 88450) was injected intraperitoneally (IP) thrice a week for two weeks following standard protocols [52].

### 2.5. Experimental Design

Normal saline (0.9% NaCl) solution was used as vehicle and for dissolving TAA and plant extract (PE). Mice were divided into control (normal saline), TAA group (TAA injected), PE group (plant extract), and treatment group TAA/PE (TAA-induced liver injury followed by oral dose of PE). The dosage plan is illustrated in Table 1. Animals were grouped in cages three days before starting the experiment. By the end of the study period, blood was collected and animals were sacrificed for liver tissue samples of all study groups. Pictures of intact liver organs were also taken for macroscopic analysis of liver morphology.

### 2.6. Liver Function Tests

Blood samples were collected using a cardiac puncture method. A sterile 3 mL syringe was used for blood collection from each mouse individually, and isolated blood was immediately transferred to nonheparinized tubes. Serum was separated from the blood by centrifugation at 4000 ×g for 10 minutes at 4°C and was used for liver function analysis, that is, alanine amino transferase (ALT), aspartate aminotransferase (AST), alkaline phosphatase (ALP), gamma-glutamyl transferase (*γ*-GT), total protein, albumin, and globulin by using ready-to-use Randox R&D kits. Serum samples were thawed and run in triplicates in 96-well reader plates according to the manufacturer's instructions.

### 2.7. Histological Examination

Isolated liver tissues were fixed in 4% paraformaldehyde and further processed for histological analysis. The 5 *μ*m thick tissue sections were stained with hematoxylin-eosin (H&E) and Periodic acid-Schiff (PAS) (Sigma-Aldrich; catalogue number 395B) reagents to assess the pathological state of liver tissues. A Nikon eclipse microscope (model TS100 fitted with a DSL3 camera) was used to capture the images of stained sections. *In situ* direct DNA fragmentation TUNEL (terminal deoxynucleotidyl transferase dUTP nick end labeling) assay staining (Abcam's TUNEL Assay Kit catalogue number ab66108) was also performed to visualize nicks in DNA strands following the kit protocol. A fluorescent microscope was used to visualize the TUNEL-positive cells.

### 2.8. RNA Extraction and cDNA Synthesis

Total mRNA was isolated from liver tissues of all study groups using Hybrid-R™ RNA purification kit (catalogue number 305-101) following the standard kit protocol. All RNA extraction experiments were performed at 4°C. Isolated RNA was resuspended in 50 *μ*L sterile water and stored at −80°C until use. 2 *μ*g RNA per sample was reverse transcribed into cDNA using Thermo Scientific RevertAid First Strand Synthesis Kit (catalogue number K1622) following the kit's instructions. cDNA samples were stored at −20°C.

### 2.9. Qualitative and Quantitative PCR Analysis

The 50 ng cDNA was used to perform qualitative PCR analysis of specific genes using primers given in Table 2. PCR products were run on 2% agarose gel, and PCR bands were observed under a UV illuminator. Pictures of gels were taken for densitometric analyses of PCR bands. Quantitative RT-PCR (PikoReal™ Real-Time PCR System catalogue number TCR0096) was performed using 25 ng cDNA. Maxima SYBR Green (catalogue number k0251) was used for master mix preparation. The PCR profile was set using PikoReal software 2.2 which was as follows: initial denaturation at 95°C and 40 cycles of 95°C, 57°C, and 72°C followed by elongation at 72°C and termination at 20°C. The expression level of genes was normalized with the housekeeping gene, *β*-actin, in the same tissue samples.

### 2.10. Statistical Analyses

One-way analysis of variance (ANOVA) with Tukey's posttest was performed to detect the significance between all study groups. Results were expressed as mean ± standard error of mean (SEM) of obtained values. For analysis, the probability value (*p*) < 0.005 was considered statistically significant. All statistical analyses were performed using GraphPad Prism version 5.00 for Windows, GraphPad Software.

## 3. Results

### 3.1. Acute Toxicity

The acute toxicity test was crucial for the determination of plant extract test dose and safety. All the tested doses of the plant extract proved safe showing no signs of behavioral changes and morbidity with an LD_50_ value of 4 g/kg body weight of mice as reported earlier [37]. This value was considered the maximum nonlethal dose (MNLD) as described elsewhere [53]. Dose selection was based on the MNLD value using a less than 1/10 of MNLD.

### 3.2. *Fagonia indica* Improves Liver Function Tests

**Figure 1:**
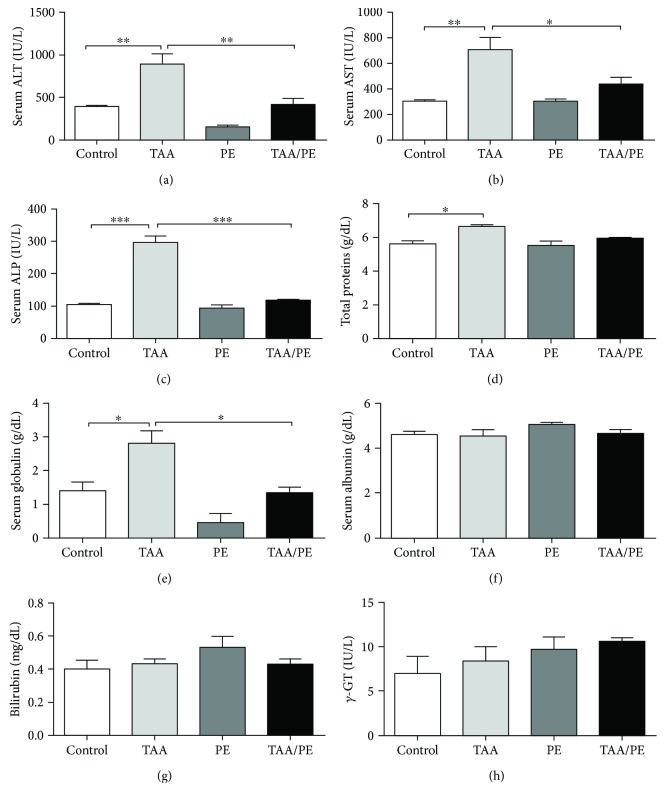
Serum biochemistry of liver injury mouse model treated with *Fagonia indica*. (a) ALT, (b) AST, (c) ALP, (d) total protein, (e) globulin, (f) albumin, (g) bilirubin, and (h) *γ*-GT levels showing significantly positive effects of plant extract in a liver injury mouse model. Statistical analysis was performed using one-way ANOVA with Tukey's posttest (^∗^*p* < 0.05, ^∗∗^*p* < 0.01, and ^∗∗∗^*p* < 0.001).

Serum biochemical analysis is an important indicator of liver function. Significant increased levels of serum ALT (*p* < 0.01), AST (*p* < 0.05), ALP (*p* < 0.001), total proteins (*p* < 0.05), and globulin (*p* < 0.05) were observed in the TAA group compared to the control depicting a successful establishment of a hepatic injury mouse model. The plant extract did not significantly alter the serum levels of ALT, AST, ALP, total protein, and globulin compared to the control. On the other hand, the TAA/PE group fed with the plant extract presented a significant decrease in serum levels of ALT (58%), AST (62%), ALP (34%), and globulin (50%) compared to the TAA group illustrating the recovery of liver function. Of note, these levels were much similar to PE and control groups (Figure 1).

### 3.3. *Fagonia indica* Repairs Liver Tissue Injury in a Mouse Model

**Figure 2:**
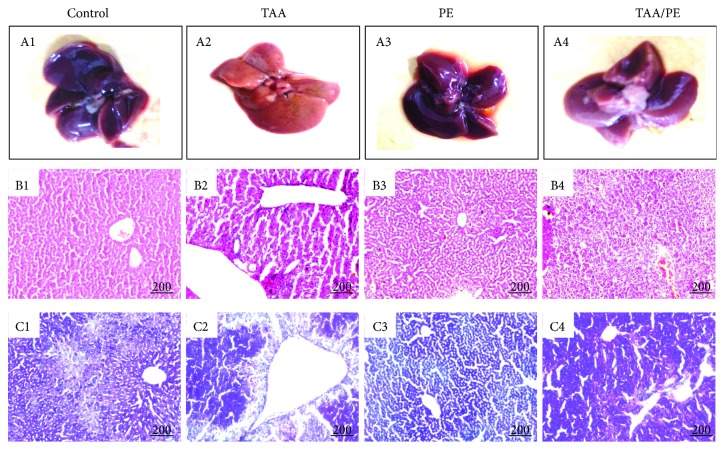
Histopathological changes of mouse liver tissues showing recovery of injury after treatment with *Fagonia indica*. Liver organs and hepatic tissue sections of the study groups were analyzed for gross morphology and histopathological alterations, respectively. (a) Gross morphology: (A1) control, (A2) TAA, (A3) PE, and (A4) TAA/PE. (b) H&E staining: (B1) control, (B2) TAA, (B3) PE, and (B4) TAA/PE. (c) PAS staining: (C1) control, (C2) TAA, (C3) PE, and (C4) TAA/PE. Histology shows repair of hepatic structure towards normal architecture due to exposure with plant extract showing injury repair activity of *Fagonia indica*.

Morphological analysis of the liver at macroscopic level indicated a reversal of liver architecture towards normal in the TAA/PE group compared to the TAA group (Figure 2(a)). In addition, histological analysis of the liver stained with H&E further elaborated the reduction of TAA-induced liver injury with the plant extract. The TAA group showed massive destruction of hepatocyte structures, increased necrosis, macrophage infiltration in the perivenular zone, tissue degeneration, and mononuclear cells in inflammatory collections. In contrast, regular hepatocyte structure, much reduced ballooning and tissue degeneration, and decreased necrotic activity were observed in the TAA/PE group (Figure 2(b)). PAS staining further strengthens our theory as results were very similar to H&E staining (Figure 2(c)).

### 3.4. *Fagonia indica* Prevents Cell Death through Inhibition of DNA Damage

**Figure 3:**
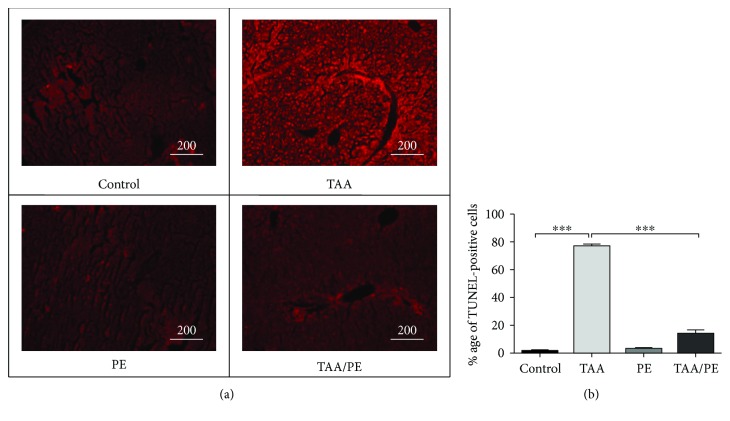
TUNEL assay staining of mouse liver tissues showing recovery of injury after treatment with *Fagonia indica*. (a) TUNEL assay staining indicates the reduction of labelled apoptotic cells in the mouse group treated with *Fagonia indica* compared to the TAA group with induced liver injury. Images shown are representative of at least three replicates. (b) Percentages of TUNEL-positive (apoptotic) cells determined by using at least six such fields of view per sample and three replicates. Statistical analysis was performed using one-way ANOVA with Tukey's posttest. Data presented as mean ± SEM (^∗∗∗^*p* 0.001).

TUNEL assay results showed a significantly high number of TUNEL-positive cells in the TAA group (*p* value <0.001). In comparison, an almost negligible number of TUNEL-positive cells for DNA breaks were observed in control PE and TAA/PE groups (*p*value < 0.001) (Figure 3(a)). Analysis of TUNEL assay data further showed a significant reduction in DNA damage in the TAA/PE group by about 17% compared to 78% observed in the TAA group (Figure 3(b)).

### 3.5. Expression Regulation of Proinflammatory, Fibrosis, and Hepatic Markers

**Figure 4:**
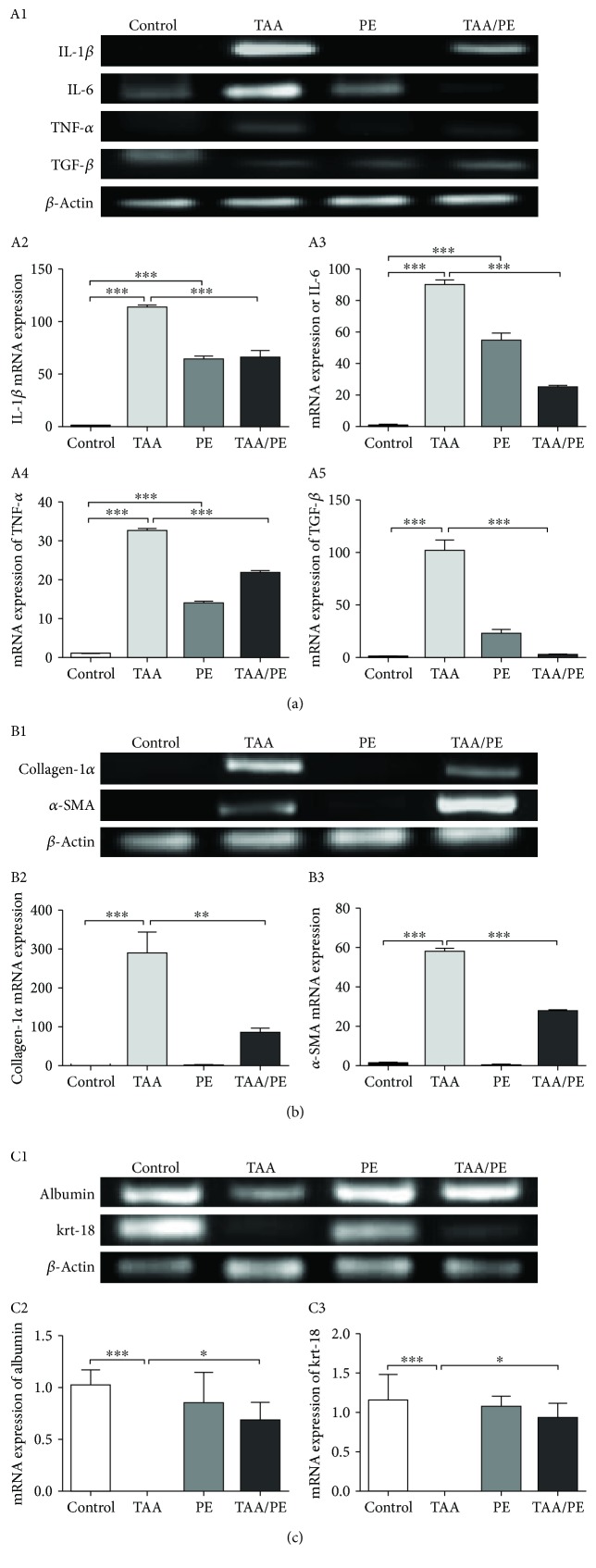
Gene expression analysis of proinflammatory cytokines and hepatic markers with or without injury after treatment with *Fagonia indica*. (a) Proinflammatory cytokines: (A1) gel electrophoresis of conventional PCR products performed for IL-1*β*, IL-6, TNF-*α*, and TGF-*β*. qRT-PCR expression analysis to quantify the changes in mRNA expressions of (A2) IL-1*β*, (A3) IL-6, (A4) TNF-*α*, and (A5) TGF-*β*. (b) Hepatic injury markers: (B1) gel electrophoresis of conventional PCR products performed for col-1*α* and *α*-SMA. qRT-PCR expression analysis to quantify the changes in mRNA expressions of (B2) col-1*α* and (B3) *α*-SMA. (c) Normal hepatic markers: (C1) gel electrophoresis of conventional PCR products performed for albumin and krt-18. qRT-PCR expression analysis to quantify the changes in mRNA expressions of (C2) albumin and (C3) krt-18. Data was normalized with *β*-actin as housekeeping control. Graphs presented show fold change in gene expression of candidate genes as mean ± SEM (^∗^*p* < 0.05, ^∗∗^*p* < 0.01, and ^∗∗∗^*p* < 0.001). Statistical analysis was performed using one-way ANOVA with Tukey's posttest.

Expression analysis with both qualitative and quantitative RT-PCR results showed significant upregulation of proinflammatory markers IL-1*β* (99.2%), IL-6 (90%), TNF-*α* (31%), and TGF-*β* (98%) in the TAA group (*p*value < 0.001) compared to the control group. However, when the TAA group was treated with PE, the expression of these genes was significantly reduced in TAA/PE mice (*p*value < 0.001) compared to TAA mice showing 48%, 63.9%, 27%, and 97% decrease, respectively (Figure 4(a)).

Similarly, expression of fibrosis markers collagen-1*α* (col-1*α*) and *α*-smooth muscle actin (*α*-SMA) was high in TAA mice compared to control mice with a probability value < 0.001, whereas, on treatment with a *Fagonia indica* extract, their expression was reduced by almost 50% in the TAA/PE group compared to TAA (Figure 4(b)).

In contrast, expression regulation of hepatocyte markers such as albumin and krt-18 was augmented in the TAA/PE group compared to downregulation in the mouse group treated with TAA. In conclusion, these results provide a molecular basis of reversal of hepatic injury on treatment with *Fagonia indica* extract (Figure 4(c)).

### 3.6. Expression Regulation of Innate Immunity Genes in Hepatic Injury Reversal

**Figure 5:**
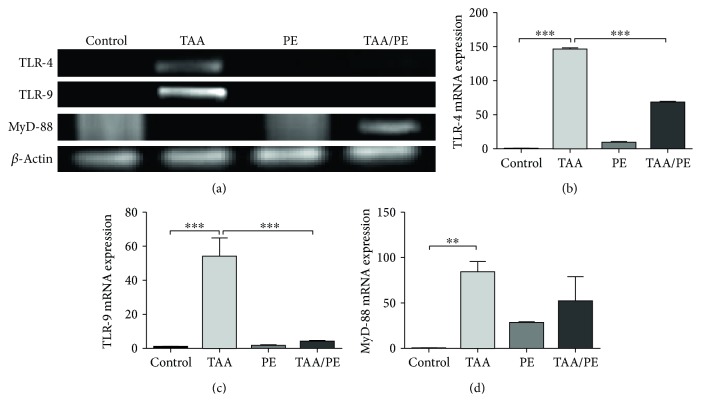
Effect of *Fagonia indica* on expression regulation of immune regulatory genes: mRNA expression of profibrotic and immune regulatory genes was analyzed in study groups. (a) Conventional PCR-amplified products of TLR-4, TLR-9, and MyD-88 genes run on agarose gel. qRT-PCR expression analysis to quantify the changes in mRNA expressions of (b) TLR4, (c) TLR9, and (d) MyD-88 compared with *β*-actin as an internal control. Statistical analysis was performed using one-way ANOVA with Tukey's posttest and presented as mean ± SEM (^∗∗^*p* < 0.01 and ^∗∗∗^*p* < 0.001).

RT-PCR analyses further exhibited significant alterations in expression of innate immunity genes such as TLR-4 and TLR-9 and downstream adaptor gene myeloid differentiation primary response 88 (MyD-88) in different experimental groups. Expression of these genes was significantly upregulated in TAA compared to the control group (*p* < 0.001, *p* < 0.001, and *p* < 0.01, resp.). Conversely, TAA/PE showed a significantly reduced expression of TLR-4 (51.3%) and TLR-9 (83.5%) with *p*value < 0.001. Expression of MyD-88 was reduced by about 42.7% compared to the TAA group (Figure 5).

## 4. Discussion

Medicinal plants with lesser side effects and more compatibility to body physiology render phytomedicine a comparatively safe treatment option since ancient times [54]. However, in order to establish potentially new pharmaceutical compounds, a comprehensive knowledge of the medicinal plants and purified components in terms of mechanism of action, active compounds, and molecular pathways involved is of immense importance. Many natural products have been already investigated at genomic, proteomic, and biochemical levels during the last decade [55, 56]. The current study establishes the hepatic injury reversal role of a medicinal plant, *Fagonia indica*, in a TAA-induced liver injury mouse model. The study also highlights the regulation of inflammatory and immune regulatory pathways in injury reversal effects of the plant.

Serum biochemistry is an important parameter for the diagnosis of liver diseases and for the assessment of the degree of liver damage [57]. Plasma levels of liver enzymes such as ALT, AST, and ALP, which are known hallmarks of TAA toxicity, are increased [58, 59]. Similar results were observed in the present study where the TAA group showed considerable elevations in serum levels of ALT, AST, ALP, total proteins, and globulin compared to the control in accordance with previous findings [60]. Noteworthily, an increase in serum globulin results from inflammation, infection, tissue necrosis, and stress. [61]. In contrast, a significant recovery of hepatic damage after treatment with *Fagonia indica* was evident from decreased plasma levels of hepatic enzymes and recovered hepatic architecture. These results are in agreement with previous findings representing the hepatoprotective activity of *Fagonia indica* [37]. Similarly, many studies have suggested the hepatoprotective role of medicinal plants in a TAA injury model [62]. Moustafa et al. [63] also reported *Coriandrum sativum* to restore normal hepatic structure and function, whereas Talluri et al. [64] proposed a restoration of hepatic physiology by *Balanites roxburghii* via inhibiting TAA toxicity.

DNA damage assessment further indicates that the plant extract plays a significant role in the retrieval of normal liver structure and function through alleviation of DNA damage. This might be attributed to the antioxidant activity of the plant against oxidative DNA damage. Several previous reports have claimed prevention of oxidative DNA breakdown by various phytomedicinal plant extracts and their constituents owing to their antioxidant properties [65–67].

Acute exposure to a hepatotoxicant leads to an upregulation of proinflammatory markers within a few hours [68]. Significant downregulation of inflammatory cytokines (IL-6, IL-1*β*, TNF-*α*, and TGF-*β*) and liver injury markers along with upregulation of normal hepatic function markers (albumin and keratin-18) further strengthens the restoration of normal liver function by *Fagonia indica*. Inhibition of proinflammatory gene expression is an important measure in detecting hepatic recovery from injury and insults [69]. Under pathological conditions, IL-6 synthesis and secretion are induced during inflammation such as upon stimulation of cells by interleukin-1 (IL-1) or TNF-*α* [70]. These inflammatory cytokines together with various growth factors are released by immune cells and play an important role in the activation of quiescent HSCs. Activated HSCs secrete an extracellular matrix, mainly collagen I, III, and IV [71, 72]. Overall, induction of TNF-*α* and TGF-*β* in Kupffer cells leads to HSC activation resulting in an upregulation of fibrosis markers *α*-SMA and col-1*α* [73]. Studies have reported reversal of liver injury via regulation of proinflammatory genes by medicinal plants such as *Aspalathus linearis* [68] and *Cynara scolymus* L. [74]. Natural products such as crocin isolated from *Crocus sativus* have been proved significantly effective in ameliorating liver injury via downregulating markers of proinflammation and fibrosis [75].

Decrease in serum globulin level coupled with expression regulation of components of immune system proposes the immune-modulatory role of *Fagonia indica.* In this regard, expression regulation of components of the innate immune system, TLR-4 and TLR-9 [6], was analyzed. Downregulation of these genes in response to plant extract treatment was suggestive of a potential immune regulatory activity of *Fagonia indica*. These results were in agreement with previous findings [76]. TLR-4 signaling involves two downstream adaptor molecule pathways: an MyD-88-dependent pathway and a TRIF- (TIR domain-containing adaptor-inducing IFN-*β*-) dependent pathway [77]. Dual signaling of these two pathways is therefore crucial for maximal TLR-4 activity [78, 79]. Previous studies have reported the MyD-88 independent role of TLR-4 in alcoholic liver disease [80]. The unaltered expression of MyD-88 after treatment with plant extract suggests that injury reversal with *Fagonia indica* occurs through an MyD-88-independent TLR-4 signaling pathway. Previously, a study has reported the liver injury reversal effect of curcumin through regulation of TLR-2, TLR-4, and TLR-9 [81].

## 5. Conclusion

Together, these findings clearly suggest that *Fagonia indica* extract has a strong hepatoprotective activity through inhibition of inflammatory and immune regulatory pathways. The current study proposes the use of a medicinal plant, *Fagonia indica*, as a hepatoprotective agent and also highlights the TLR pathway as an important therapeutic target in liver diseases.

## Figures and Tables

**Table 1:** Animal groups and treatment plan.

Animal group	Dose administration plan	Method
Control	Normal sterile saline solution (1 mL/kg body weight)	5 IPs, 15 ad libitum doses
TAA	TAA in saline solution (100 mg/kg body weight) 3 times a week	5IPs
PE	Plant extract (150 mg/kg body weight) 6 days a week	15 ad libitum doses
TAA/PE	TAA administration followed by PE	5IPs/15 ad libitum doses

TAA: thioacetamide; PE: plant extract; IP: intraperitoneal injections.

**Table 2:** Primers used in this study.

Mouse primer	Product lengths	Sequence
TLR4 F	268 bp	5′-TCC CTG CAT AGA GGT AGT TC-3′
TLR4 R		5′-ACT CTG GAT AGG GTT TCC TG-3′
TLR9 F	310 bp	5′-GCCTCCGAGACAACTACCTA-3′
TLR9 R		5′-CTGCTGACATCCAGTTTCTG-3′
MyD-88 F	223 bp	5′-GGCATCTGCATATGTGTGTT-3′
MyD-88 R		5′-CCCAGGCTGACCTTAAACTA-3′
Collagen 1 F	301 bp	5′-TGA GTC AGC AGA TTG AGA AC-3′
Collagen 1 R		5′-TAC TCG AAC GGG AAT CCA TC-3′
IL-1*β* F	268 bp	5′-GTA CAT CAG CAC CTC ACA AG-3′
IL-1*β* R		5′-CAC AGG CTC TCT TTG AAC AG-3′
*α*-SMA F	418 bp	5′-GCA TCC ACG AAA CCA CCT A-3′
*α*-SMA R		5′-CAC GAG TAA CAA ATC AAA GC-3′
TNF-*α* F	67 bp	5′-CTC CAG GCG GTG CCT ATG T-3′
TNF-*α* R		5′-GAA GAG CGT GGT GGC CC-3′
TGF-*β* F	69 bp	5′-CCC GAA GCG GAC TAC TAT GC-3′
TGF-*β* R		5′-ATA GAT GGC GTT GTT GCG GT-3′
IL-6 F	73 bp	5′-CCA GAA ACC GCT ATG AAG TTC C-3′
IL-6 R		5′-TCA CCA GCA TCA GTC CCA AG-3′
*β*-Actin F	62 bp	5′-GAA GTC CCT CAC CCT CCC AA-3′
*β*-Actin R		5′-GGC ATG GAC GCG ACC AT-3′
Krt-18F	150 bp	5′-GAAGAGCCTGGAAACTGAGAAC-3′
Krt-18R		5′-TTGTCCACAGAATTCGCAAAGA-3′
Albumin F	222 bp	5′-GAAGTGCTCCAGTATGCAGAAG-3′
Albumin R		5′-GAGATAGTCGCCTGGTTTTCAC-3′

## Data Availability

The data used to support the findings of this study are available from the corresponding author upon request.
